# Anatomy of a Rescue: Explantation and Reconstruction After Complex Endovascular Aortic Repair—A Systematic Review of Case Reports

**DOI:** 10.3390/medsci14050574

**Published:** 2026-09-16

**Authors:** Luis O. Bobadilla-Rosado, Luis H. Arzola-Flores, Carlos Arturo Hinojosa-Becerril, Maria José Sanabrais-López, Alberto Azueta-Cervera, Fabiola Velazquez-Castro, Osvaldo Huchim-Mendez, Nina Mendez-Domínguez

**Affiliations:** 1 Instituto Nacional de Ciencias Médicas y Nutrición Salvador Zubirán, Mexico City 14080, Mexico; luis.arzolaf@incmnsz.mx (L.H.A.-F.); carlos.a.hinojosa@gmail.com (C.A.H.-B.); 2 Hospital Regional Elvia Carrillo Puerto, ISSSTE, Mérida 97001, Mexico; mariajosesanabrais11@gmail.com; 3 Hospital Regional de Alta Especialidad de la Península de Yucatán, IMSS-Bienestar, Mérida 97300, Mexico; albertoazueta01@gmail.com (A.A.-C.); faby.vel04@gmail.com (F.V.-C.); 4 School of Medicine, Universidad Anáhuac Mayab, Mérida 97310, Mexico; ohuchim@anahuac.mx

**Keywords:** endovascular aneurysm repair, device removal, blood vessel prosthesis, prosthesis-related infections, reoperation, vascular surgical procedures

## Abstract

**Background:** Explantation after chimney, fenestrated, or branched endovascular aortic repair is technically demanding because major arterial branches are incorporated into the device. Objective: To identify recurring principles of operative decision-making for partial or complete explantation, with an emphasis on infection extent, incorporated branch management, reconstruction, and staging. **Methods:** PubMed/MEDLINE and Scopus were searched from inception through 21 August 2026 for patient-level reports of surgical explantation after complex endovascular aortic repair. Two reviewers independently selected studies, extracted data, and assessed methodological quality with the Joanna Briggs Institute checklist and reporting completeness with CARE. Findings were synthesized descriptively; the protocol was registered in PROSPERO (CRD420261412667). **Results:** Ten single-patient reports published from 2015 to 2025 were included. Patients were aged 51–78 years (median, 73), and nine were men. Infection prompted eight explantations; renal chimney-stent thrombosis with type Ia endoleak and persistent type II endoleak with sac enlargement accounted for two mechanical failures. Computed tomography or computed tomography angiography was reported in all cases and FDG-PET/CT in five. Eight patients underwent complete and two partial explantations. Nine survived the index hospitalization. No recurrent infection was reported during the available, heterogeneous follow-up of 3–48 months. **Conclusions:** These selected reports identify anatomy- and source-control considerations that may guide operative planning, but they do not establish comparative effectiveness, procedural safety, or population-level outcomes.

## 1. Introduction

Fenestrated, branched, and chimney endovascular techniques have extended aortic repair to patients with juxtarenal, pararenal, thoracoabdominal, and aortic arch aneurysms whose anatomy is unsuitable for standard endovascular treatment. Contemporary multicenter evidence supports their effectiveness. In a prospective cohort of 1109 patients undergoing fenestrated-branched endovascular aortic repair (bEVAR-fEVAR) for thoracoabdominal aortic aneurysms, early mortality was 2.7%, whereas the five-year cumulative incidence of aortic-related mortality and rupture were 3.8% and 2.7%, respectively. Nevertheless, 40.3% of patients required a secondary intervention within five years [1].

Earlier long-term experience with fenestrated endovascular repair found that 37% of patients underwent at least one reintervention, with 68% of these procedures prompted by abnormalities detected during routine surveillance [2]. Although many complications can be managed with additional endovascular procedures, endograft infection, aortoenteric fistulization, persistent endoleak with sac expansion, branch occlusion, device migration or separation, and progressive pseudoaneurysm formation may ultimately require open conversion with partial or complete endograft explantation and in selected cases, extensive debridement and prolonged antimicrobial therapy may become necessary [3,4].

Explantation after chimney (chEVAR), bEVAR, or fEVAR is particularly challenging because the endograft may incorporate renal, visceral, or supra-aortic vessels. Device removal may therefore require complex exposure, control of multiple aortic branches or reconstruction of target vessels. These technical and anatomical circumstances are seldom described in sufficient detail in conventional outcome studies, where several distinct complications and reinterventions may be combined into aggregate endpoints.

Published evidence on complex endograft explantation is sparse and consists predominantly of individual case reports. Although such reports cannot estimate incidence, comparative effectiveness, or procedural safety, systematic case-level appraisal can preserve the anatomical and technical detail that is often lost when heterogeneous reinterventions are aggregated in larger series [5,6,7]. Its principal value is therefore to identify recurring considerations in operative decision-making rather than to pool outcomes.

The primary objective of this systematic review was to identify recurring operative decision principles in published cases of partial or complete explantation after chEVAR, bEVAR, or fEVAR, specifically the extent of infection or structural failure, feasibility of partial versus complete removal, management of incorporated renal, visceral, or supra-aortic branches, selection of reconstruction material, and use of staged versus single-stage procedures. We secondarily described presentation, diagnostic and microbiological findings, antimicrobial treatment, postoperative complications, survival, and follow-up.

## 2. Materials and Methods

### 2.1. Protocol and Reporting Framework

This systematic review was conducted according to a prospectively developed protocol and reported in accordance with the Preferred Reporting Items for Systematic Reviews and Meta-Analyses 2020 statement [8]. The protocol was registered in PROSPERO on 15 July 2026 (CRD420261412667). Because the available evidence consisted of individual case reports without comparator groups, denominators, or sufficiently homogeneous outcomes, the review was designed for descriptive and narrative synthesis rather than meta-analysis.

### 2.2. Eligibility Criteria

We included published case reports and case series describing patients who underwent partial or complete surgical explantation of an aortic endograft previously implanted during complex endovascular aortic repair. Eligible index procedures included chimney endovascular aneurysm repair (chEVAR), fenestrated endovascular aneurysm repair (fEVAR), branched endovascular aneurysm repair (bEVAR), and branched thoracic endovascular aortic repair when the device incorporated visceral and/or supra-aortic branches.

Reports were eligible if they provided patient-level information on the indication for explantation, clinical presentation, diagnostic assessment, operative procedure, or postoperative outcome. For case series, inclusion required that information about eligible patients could be extracted separately. Only published studies were included, in accordance with the registered protocol.

We excluded reports involving only standard infrarenal EVAR or conventional thoracic endovascular aortic repair without supra-aortic branch incorporation. We also excluded patients managed exclusively with conservative treatment or endovascular reintervention without surgical graft removal. Publications in English or Spanish were eligible, with no restriction on publication year.

### 2.3. Information Sources and Search Strategy

PubMed/MEDLINE and Scopus were searched from database inception through 21 August 2026. The strategy combined controlled vocabulary, where available, and free-text terms for complex endovascular repair (fenestrated, branched, chimney, fEVAR, bEVAR, and chEVAR) with terms for device removal, explantation, and open conversion. No publication-year restriction was applied. Complete database-specific search strings, search dates, and record counts are provided in Supplementary File S1.

### 2.4. Study Selection and Data Extraction

**Figure 1:**
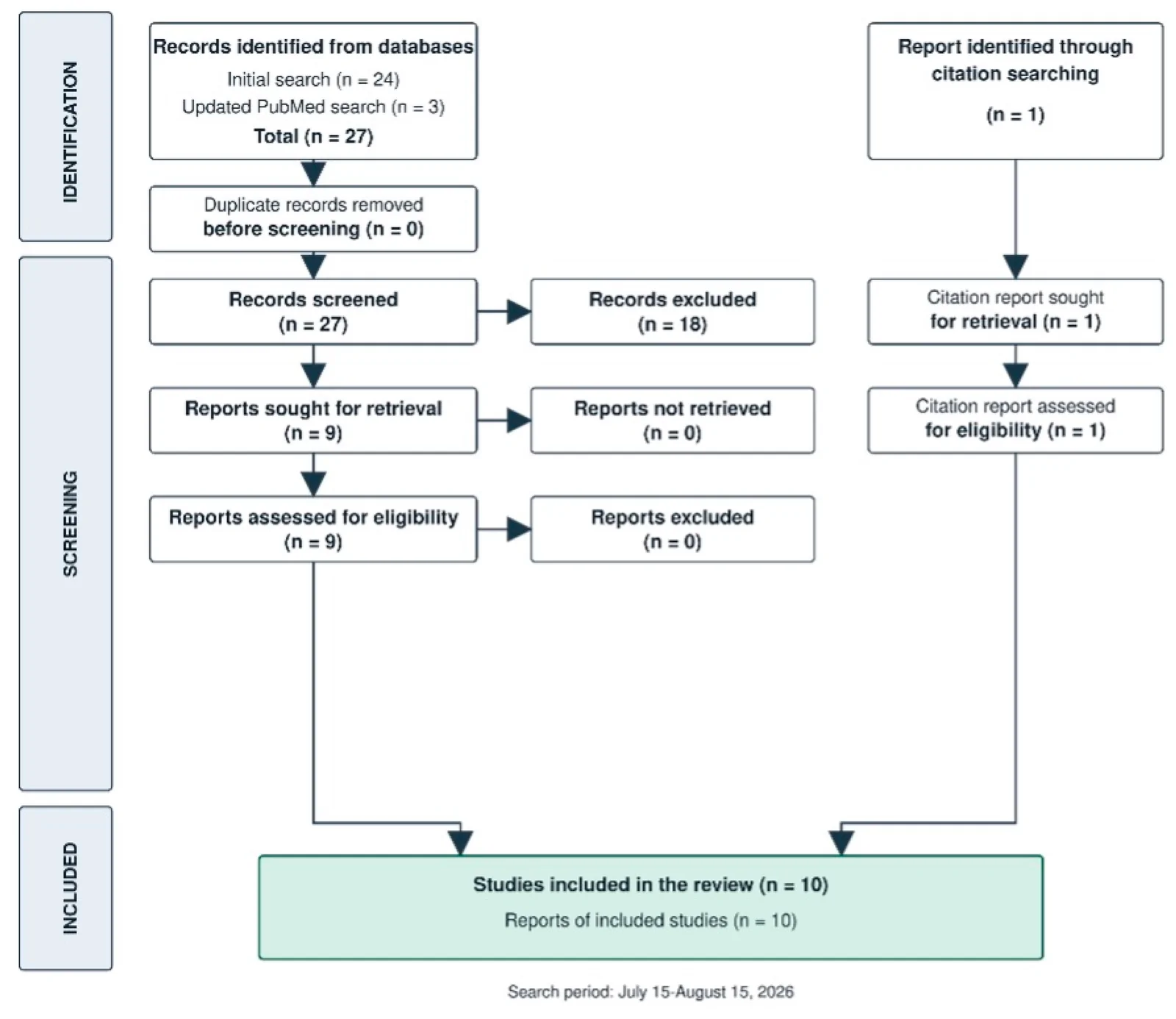
PRISMA flow diagram regarding the selection process for the individual case reports.

Records from both databases were combined, and duplicates were identified using title, author, year, and DOI information and removed before screening. Two reviewers independently screened titles and abstracts and then assessed potentially eligible full texts. Full-text reports were excluded when they described standard EVAR or thoracic EVAR without incorporated branches, did not include surgical graft removal, lacked separately extractable patient-level data, or did not meet the language criteria. Case series were eligible only when data for an individual qualifying patient could be extracted separately. Reasons and counts for full-text exclusion are shown in Figure 1. The same reviewers independently extracted clinical, imaging, microbiological, operative, antimicrobial, and outcome data; disagreements at selection, extraction, or appraisal were resolved by consensus.

#### 2.4.1. Methodological Quality, Reporting Completeness, and Missing Data

Two reviewers independently assessed methodological quality with the Joanna Briggs Institute Critical Appraisal Checklist for Case Reports [9] and reporting completeness with the CARE checklist [10]. CARE was treated as a reporting guideline rather than a risk-of-bias instrument. Neither assessment was used to exclude or numerically weight reports; instead, both were used to qualify the certainty and interpretability of case-level observations. Unreported information was coded as not reported and was not interpreted as an absence of a complication, microorganism, treatment, or recurrent infection. Appraisal disagreements were resolved by consensus.

#### 2.4.2. Data Synthesis

Clinical and technical characteristics were tabulated at the patient level and synthesized descriptively and narratively according to recommendations for case reports and case series [11]. Because there were no comparator groups, denominators, or sufficiently homogeneous outcomes, no meta-analysis or comparative-effect estimate was calculated. Recurring case-level patterns were treated as hypothesis-generating observations and were distinguished from recommendations supported by independent guidelines or larger vascular graft infection series. Additional methodological details are provided in Supplementary File S1.

### 2.5. Ethical Considerations

The review used information from previously published reports and did not involve direct contact with patients or access to identifiable primary clinical records. Therefore, institutional ethics approval and additional informed consent were not required for the systematic review. Consent reporting in each original case report was considered during the CARE assessment.

## 3. Results

### 3.1. Publications Included

The 10 included publications were single-patient reports published between 2015 and 2025 and originated from five countries: six from the United States and one each from Belgium, France, Italy, and Switzerland [12,13,14,15,16,17,18,19,20,21]. Eight were published from 2021 onward and five in 2025. This distribution describes the available literature only; without denominators for complex endovascular procedures, surveillance duration, or center-level reporting, it cannot establish that explantation or infection rates have increased over time.

### 3.2. Methodological Quality and Reporting Completeness

The JBI and CARE assessments showed variable reporting across domains, particularly for longitudinal follow-up, adverse events, antimicrobial regimens, and recurrent infection. The appraisals were therefore used to identify limits in interpretation rather than to rank or weight cases. Given the small evidence base, it was not possible to determine whether less completely reported cases contributed disproportionately to any observed pattern. Accordingly, missing items are reported as unavailable, and no conclusion of clinical absence was drawn from non-reporting. Detailed item-level assessments are provided in Supplementary File S1.

### 3.3. Patient Characteristics and Clinical Course

The ten patients were aged 51–78 years, with a median age of 73 years, and nine were men. Most were older patients with substantial cardiovascular or respiratory comorbidities. The only woman was the youngest patient, reported by Catalano et al. [17]; she had autoimmune disease, chronic immunosuppression, and aortitis associated with *Clostridium septicum* bacteraemia. Other patient-specific circumstances such as refusal of blood transfusions directly affected treatment planning.

Eight patients underwent abdominal or thoracoabdominal repair incorporating renal or visceral vessels through fenestrations, branches, or chimney stents. The other two had branched thoracic endografts involving the aortic arch and the left subclavian artery. Thus, all explantations involved devices connected to major arterial branches, but the anatomical requirements differed. Abdominal and thoracoabdominal repairs required renal and visceral protection or reconstruction, whereas the arch cases required cardiopulmonary bypass, hypothermia, and circulatory arrest.

Endograft infection was the primary indication for explantation in eight patients. Clinical presentation varied and was often nonspecific. Abdominal infections commonly presented with abdominal or back pain, malaise, anorexia, weight loss, and increased inflammatory markers. Fever was not consistently present even in cases with extensive infection and/or enteric fistulisation. In thoracic cases, recurrent chest pain, rapid pseudoaneurysm progression and hemoptysis associated with a suspected aorto-esophageal fistula were reported.

The interval from endovascular repair to explantation was highly variable, ranging from 3 months [17] to seven years [16]. Early complications included endograft infection reported in 2 cases [13,17] and chimney thrombosis [12].

The infectious course was more destructive when abscesses, fistulas, or intestinal erosion were present. In 3 cases, an aortoenteric fistula was reported [13,14,19]. Reported organisms included Moraxella osloensis, Staphylococcus aureus, Escherichia coli, Actinomyces odontolyticus, Listeria monocytogenes, and Parvimonas micra. Hanif et al. [19] reported the most complex microbiological profile, including several bacterial species and Saccharomyces cerevisiae.

The two non-infectious cases followed different courses: one consisted of a persistent type IIb endoleak with aneurysm sac enlargement from 6.2 to 10 cm despite four endovascular reinterventions and the other described early mechanical failure of chEVAR, with compression and bilateral thrombosis of the renal chimney stents, recurrent type Ia endoleak, severe acute kidney injury, and hyperkalemia.

### 3.4. Imaging Studies and Route for Hospital Admission

Computed tomography (CT) or computed tomography angiography (CTA) was reported in all 10 cases and represented the principal modality for diagnosis, anatomical characterization, and operative planning. FDG-PET/CT was used in five cases, primarily to confirm or delineate suspected infection, whereas duplex ultrasonography, catheter angiography, and labeled white blood cell scintigraphy were used selectively. Nine patients underwent imaging because of new symptoms, clinical deterioration, readmission, or urgent referral. Only two reports explicitly documented presentation to the emergency department, while only one complication (the persistent type II endoleak reported by Steadman et al. [15]) was initially identified during scheduled imaging surveillance.

Preoperative imaging identified most major topographical constraints, including graft extent, visceral or supra-aortic branch incorporation, proximal fixation level, aneurysm sac enlargement, perigraft collections, and the relationship between the infected field and adjacent digestive structures. It was also instrumental in identifying apparently uninvolved graft segments that could potentially be preserved. Imaging findings are presented in Table 1.

### 3.5. Other Tests

Additional laboratory and clinical investigations were inconsistently reported. C-reactive protein and leukocyte count were the most frequently reported inflammatory biomarkers, although normal leukocyte counts and negative blood cultures did not exclude endograft infection. Microbiological confirmation was obtained through blood cultures, percutaneous drainage, or deep intraoperative sampling, and operative specimens occasionally identified additional organisms that prompted antimicrobial modification. When a test, organism, treatment, or complication was not described, it was treated as not reported rather than absent.

### 3.6. Therapeutic Approach

Complete explantation was reported in eight patients and partial explantation in two. In the non-infectious case described by Steadman et al. [15], the proximal fenestrated component and iliac limbs were retained because no type I or III endoleak was identified and the visceral segment remained stable, thereby avoiding supraceliac clamping and visceral reimplantation. In the infected case described by Manunga et al. [16], infection was reported as confined to the infrarenal aorta; after radical debridement, the retained fenestrated cuff served as a sewing ring for an in situ rifampicin-soaked Dacron graft with complete omental coverage. These are selected case-level strategies and do not establish the comparative safety of partial removal.

Operative duration was reported in only three cases and ranged from 6 h 8 min to 8.5 h. Complete explantation frequently required thoracoabdominal or retroperitoneal exposure, proximal control above the visceral vessels, extensive debridement, and reconstruction of renal, mesenteric, or supra-aortic branches. The two arch cases required cardiopulmonary bypass, deep hypothermia, circulatory arrest, and supra-aortic revascularization. Temporary abdominal or thoracoabdominal closure followed by planned re-exploration was used after several extensive procedures; this should be distinguished from staged revascularization and explantation. Table 2 summarizes access, branch and organ-protection strategies, reconstruction, staging intervals, operative duration, and major resource requirements for all 10 cases while Figure S1 provides a schematic representation.

The included reports described complete removal when infection was diffused or accompanied by sepsis, fistulization, purulence, progressive pseudoaneurysm, or major mechanical failure. Eight cases used in situ reconstruction, with autologous femoral or saphenous vein, antimicrobial or rifampicin-soaked Dacron, cryopreserved allograft, bovine pericardial tube, or biosynthetic conduits. This variation likely reflects contamination, anatomical requirements, material availability, and institutional experience; the case reports do not support preference for a specific conduit.

Two abdominal cases used staged extra-anatomical revascularization [19,21]. Hanif et al. performed a right axillobifemoral bypass followed 48 h later by explantation, debridement, and renal and visceral reconstruction with a cryopreserved allograft [19]. Bugna et al. performed a rifampicin-soaked axillobifemoral bypass followed 24 h later by explantation and multibranch reconstruction with a cryopreserved aortoiliac allograft [21]. These reports illustrate separation of limb revascularization from contaminated-field surgery, but they do not permit comparison with single-stage in situ repair.

Renal and visceral perfusion was a central concern in the abdominal and thoracoabdominal cases. Different strategies such as thoracorenal bypass, cold renal perfusion, combined renal thrombectomy, visceral debranching, left renal to superior mesenteric and right renal bypass, sequential clamping and left or right renal artery reimplantation were used.

The two thoracic cases required distinct strategies. Catalano et al. used posterolateral thoracotomy, cardiopulmonary bypass, deep hypothermia, circulatory arrest, and distal arch and descending thoracic reconstruction [17]. Trimarchi et al. used a two-stage procedure: carotid-subclavian bypass was performed 2 days before explantation under circulatory arrest and reconstruction with a bovine pericardial tube [18]. These cases illustrate the additional demands created by aortic arch and supra-aortic branch involvement.

### 3.7. Postoperative Course and Outcomes

Length of hospital stay ranged from 3 to 48 days (median, 18.5 days). Nine patients survived the index admission. One in-hospital death occurred on postoperative day 3 after bleeding, bowel ischemia, myocardial infarction, and multiorgan failure. These selected observations are not a survival estimate.

In three cases [13,16,21], antibiotics or drainage did not eradicate the reported infection before definitive surgery, and all three patients survived the index admission after explantation. This sequence is descriptive and does not establish that treatment timing caused the observed outcomes.

Reported postoperative complications principally involved renal, visceral, and neurological ischemia and major bleeding. They included acute kidney injury requiring temporary hemodialysis, bypass thrombosis, shock, coagulopathy, massive transfusion, transient mesenteric ischemia, and cerebellar stroke without residual neurological deficit. Non-reporting was not interpreted as absence of a complication.

No recurrent infection was reported among the nine survivors during the available and heterogeneous follow-up of 3–48 months. The longest reported follow-up was four years. These data cannot establish freedom from reinfection because follow-up and recurrence assessment were not standardized.

Across the reports, the apparent decision points were the distribution of infection or structural failure, availability of healthy aortic tissue, stability of any component considered for retention, incorporated branch anatomy, and the feasibility of in situ versus staged extra-anatomical reconstruction. These observed patterns are explored as hypothesis-generating principles in the Discussion section.

## 4. Discussion

### 4.1. Contribution and Evidence Boundaries

The main contribution of this review is not an estimate of outcome but a structured account of recurring operative decisions in rare, heterogeneous explantations. The reports combined chimney, fenestrated, thoracoabdominal-branched, and arch-branched devices; infectious and mechanical indications; and fundamentally different operative environments. Consequently, every pattern below is hypothesis-generating. Recommendations are identified separately when they derive from contemporary vascular graft and endograft infection guidance rather than from the 10 cases [4].

Nine patients survived the index hospitalization, but this observation cannot be interpreted as procedural safety or an expected survival rate. Published case reports preferentially represent technically instructive or successful procedures, omit patients not offered surgery, and provide heterogeneous follow-up [7,11]. Likewise, the concentration of reports after 2021 may reflect broader use of complex endovascular repair, delayed failures, or greater publication interest, but the absence of procedural denominators prevents inference about temporal changes in explantation or infection rates.

### 4.2. Infection Extent and Partial Versus Complete Explantation

The case-level pattern was anatomically coherent: diffuse contamination, fistulization, purulence, destructive pseudoaneurysm, or major mechanical failure accompanied complete explantation, whereas partial removal was reported only when a retained component had a stable seal, was firmly incorporated, and appeared outside the pathological field [15,16]. Contemporary ESVS guidance similarly emphasizes source control, excision of infected material when feasible, debridement, and individualized reconstruction [4]. The present cases do not establish that partial or complete removal is superior; rather, they show which anatomical and infection-related features were considered in the reported decisions.

### 4.3. Incorporated Branches, Reconstruction Material, and Staging

Management of incorporated renal, visceral, and supra-aortic branches defined the operative burden. Abdominal and thoracoabdominal cases used combinations of supraceliac control, cold renal perfusion, thrombectomy, branch reimplantation, or bypass, whereas arch cases required cardiopulmonary bypass, hypothermia, circulatory arrest, and preliminary supra-aortic revascularization. Staging separated preliminary revascularization from explantation by 24–48 h in two abdominal cases [19,21] and by 2 days in one arch case [18]. These intervals and strategies reflect individual reports and cannot identify an optimal staged approach.

Reconstruction materials also varied, including autologous veins, rifampicin-soaked Dacron, cryopreserved allograft, bovine pericardium, and biosynthetic conduits. Current guidance supports selecting in situ or extra-anatomical reconstruction according to infection extent, organism, tissue quality, anatomy, and patient fitness [4]. The present review offers no comparative basis for preferring one conduit; it instead underscores the need to match the reconstruction to the contaminated field and the required target-vessel anatomy.

### 4.4. Diagnosis, Source Control, and Antimicrobial Therapy

Diagnosis was frequently symptom-driven and could not rely on fever, leukocytosis, or blood culture alone. Computed tomography or computed tomography angiography characterized graft extent, branch incorporation, collections, gas, sac enlargement, and adjacent digestive involvement in every case. Fluorodeoxyglucose positron-emission tomography/computed tomography and labeled-leukocyte scintigraphy were used selectively when an infection or its extent remained uncertain. This multimodal approach is consistent with the MAGIC framework and ESVS guidance, which integrate clinical or operative, radiological, and laboratory evidence [4,22].

Antimicrobial therapy should accompany, not substitute for, source-control planning when explantation is feasible. Blood cultures and deep intraoperative specimens should be obtained whenever possible, with empirical broad-spectrum treatment narrowed according to organism identification and susceptibility. Contemporary ESVS guidance supports individualized duration: after complete excision and debridement, at least six weeks may be considered, whereas retained infected material, incomplete source control, or recurrent infection may require prolonged or lifelong suppressive treatment [4,23,24,25,26,27]. Isolation of Saccharomyces cerevisiae requires confirmation of invasive infection and susceptibility testing because fluconazole activity cannot be assumed [28].

### 4.5. Pathophysiology and Inflammatory Biomarkers

Infectious and mechanical failure should not be viewed as entirely independent pathways. A prosthetic surface, perigraft thrombus, endothelial and wall injury, bacterial adherence or biofilm, local tissue ischemia, and extracellular-matrix degradation may interact to sustain inflammation, thrombosis, loss of tissue integrity, fistulization, or pseudoaneurysmal change [3,4]. In the included reports, C-reactive protein and leukocyte count were the most frequently described inflammatory markers, but their inconsistent measurement and the absence of longitudinal thresholds precluded prognostic interpretation.

Evidence from other vascular beds provides a rationale for future study, not direct proof in aortic endografts. In 100 patients undergoing carotid endarterectomy or carotid artery stenting, Scalise et al. found that preprocedural neutrophil-to-lymphocyte ratio and systemic inflammation response index were independently associated with 12-month restenosis [29]. Because the anatomy, procedure, and outcome differ from complex aortic endograft failure, these findings should not be extrapolated to infection or explantation risk. They nevertheless support prospective evaluation of inflammatory and thrombo-inflammatory biomarkers as potential modifiers or surveillance markers after complex endovascular aortic repair.

### 4.6. Outcomes and Reporting Quality

No recurrent infection was reported among survivors during 3–48 months of available follow-up, but this is not equivalent to demonstrated freedom from reinfection. JBI and CARE appraisal identified variable completeness, especially for complications, antimicrobial treatment, follow-up, and recurrence ascertainment. Because missing information was coded as not reported and the reports were not weighted, no clinical inference was based on an assumed absence. Larger consecutive series or registries with standardized infection definitions, biomarker sampling, treatment reporting, and longitudinal surveillance are needed to test the patterns identified here.

## 5. Limitations

This review is limited to represent 10 highly selected single-patient reports; the absence of denominators, control groups, and consecutive experience prevents estimation of explantation incidence, prognostic factors, procedural safety, or comparative effectiveness. Publication and selection bias are likely, and substantial anatomical, clinical, and methodological heterogeneity precluded quantitative synthesis.

Important variables were incompletely or inconsistently reported, infection criteria were not standardized, prior antimicrobials may have reduced microbiological yield, and the follow-up was heterogeneous. The predominance of specialized Western centers also limits generalizability. JBI and CARE appraisal could identify reporting limitations but could not eliminate bias or support case weighting.

## 6. Conclusions

Published case reports suggest that operative planning for complex endograft explantation is driven by infection or failure extent, stability of any component considered for retention, incorporated branch anatomy, available reconstruction zones, and the need for staged revascularization.

These patterns may structure multidisciplinary planning, but they remain hypothesis-generating and do not establish the superiority, safety, or durability of any explantation or reconstruction strategy.

## Figures and Tables

**Table 1:** Imaging findings reported in the evaluation and characterization of the 10 cases.

Case	Imaging Used to Diagnose or Characterize the Complication	Route of Presentation
Scali et al., 2015 [12]	Noncontrast CT demonstrated chimney-stent compression; duplex ultrasound showed absence of renal artery flow. Previous surveillance CT documented renal perfusion and sac thrombosis.	Acute admission at a referring institution for flank pain and nausea, followed by urgent transfer. Not detected during routine follow-up.
Terry et al., 2017 [13]	CT/CTA demonstrated an inflammatory periaortic cuff, gas, and multiple intra-abdominal and psoas abscesses. Serial CT was used during conservative management.	Unscheduled readmission one month after FEVAR because of infection and persistent fever. Several subsequent readmissions occurred before explantation.
Caradu et al., 2021 [14]	CTA demonstrated a perigraft collection. FDG-PET showed focal uptake, confirmed by labeled white blood cell scintigraphy. Chest radiography and echocardiographic evaluation for endocarditis were negative.	Symptomatic presentation immediately after the latest endovascular procedure, with abdominal and back pain, weight loss, and markedly elevated inflammatory markers. The report does not explicitly state ED presentation.
Steadman et al., 2022 [15]	Serial multiphase CTA demonstrated the type II endoleak and progressive sac enlargement. Duplex ultrasound and catheter angiography were used to exclude type I or III endoleaks; abdominal aortography was also performed.	The only case clearly identified through scheduled imaging surveillance. The endoleak was first detected on follow-up CTA 16 months after the index repair.
Manunga et al., 2023 [16]	CTA showed aneurysm-wall thickening, inflammatory changes, and possible periaortic fluid. FDG-PET/CT localized uptake to the infrarenal sac. CT-guided aspiration was performed, and repeat PET supported persistent infection.	Symptom-driven presentation to an outside institution, followed by transfer to the reporting center.
Catalano et al., 2025 [17]	Serial CTA demonstrated inflammatory aortitis, rapid aortic enlargement, periaortic gas, fluid, and a proximal pseudoaneurysm. FDG-PET showed substantial uptake around the stented arch.	Mixed pathway: initially symptom-driven, followed by serial CTA during treatment. Subsequent hypertensive emergency and recurrent chest pain led to urgent intervention and later transfer for explantation.
Trimarchi et al., 2025 [18]	CT strongly suggested endograft infection and an aorto-esophageal fistula. Intraoperative esophagoscopy was used to evaluate the suspected defect.	Explicit presentation to the emergency department with hemoptysis.
Hanif et al., 2025 [19]	CT demonstrated an infected fenestrated endograft with extensive purulent material within the aneurysm sac.	Explicit presentation to the emergency department after four days of abdominal and back pain, fever, and malaise.
Panettella et al., 2025 [20]	Multislice CTA demonstrated periaortic enhancement and fat stranding. FDG-PET/CT showed intense heterogeneous uptake involving the stent graft and thrombotic material within the sac.	Clinical deterioration during rehabilitation prompted readmission and referral from an external clinic. Emergency explantation followed, but an ED presentation was not explicitly reported.
Bugna et al., 2025 [21]	PET/CT initially demonstrated a psoas abscess and periaortic inflammation. At recurrence, CTA demonstrated sac gas, periaortic inflammation, and iliac branch device occlusion. Three-dimensional CTA reconstructions were used for surgical planning.	Symptom-driven infection at 14 months and symptomatic recurrence at 29 months. The report does not explicitly identify an ED presentation.

**Table 2:** Operative access, organ protection, reconstruction, staging, and resources in the 10 included cases.

Case	Extent and Surgical Access	Aortic/Organ Protection and Reconstruction	Operative Time and Resources
Scali et al.	Complete device explantation through retroperitoneal access	Supraceliac clamping; renal thrombectomy; beveled anastomosis incorporating the SMA and right renal artery; left renal bypass; aorto-bi-iliac reconstruction	Duration and blood loss not reported. Urgent hemodialysis was required before surgery, followed by three postoperative sessions
Terry et al.	Complete explantation through thoracophrenolaparotomy at the eighth rib	Axillobifemoral bypass performed first; superficial femoral and great saphenous veins harvested; autologous in situ venous reconstruction with SMA and bilateral renal bypasses; aortoduodenal fistula closure	8.5 h. Renal ischemia times after intra-arterial cooling: 45 and 75 min; bowel ischemia: 90 min. Two bleeding revisions and subsequent bowel resection were required
Caradu et al.	Complete explantation through ninth-intercostal thoracophrenolumbotomy with circumferential phrenotomy	Thoracorenal bypass before explantation; supraceliac clamping; cold right renal perfusion; complete debridement and aortectomy; bifurcated antimicrobial graft below the SMA; right renal reimplantation	6 h 8 min. Left renal warm ischemia: 25 min; visceral warm ischemia: 30 min; right renal cold ischemia: 60 min. Temporary laparostomy, packing and definitive reconstruction/closure 72 h later
Steadman et al.	Partial infrarenal explant through midline transperitoneal laparotomy; proximal fenestrated component and iliac limbs preserved	Supraceliac and suprarenal clamps were prepared but not applied; the preserved infrarenal graft served as the clamp site; polyester interposition graft; lumbar artery ligation	Duration and blood loss not reported. SMA stent compression required intraoperative restenting and angiography. Temporary closure and second-look surgery on postoperative day 1
Manunga et al.	Partial explant through midline transperitoneal access; attempted left medial visceral rotation	Supraceliac clamping for 10 min, followed by infrarenal clamping; radical debridement; preservation of incorporated fenestrated cuff as a sewing ring; rifampicin-soaked bifurcated Dacron graft and 360° omental wrap	Duration not reported. Estimated blood loss: 2 L
Catalano et al.	Complete thoracic branched endograft explant through left posterolateral thoracotomy	Lumbar drain; cardiopulmonary bypass via descending thoracic aorta and femoral vein; cooling to 18 °C; EEG-guided circulatory arrest with retrograde perfusion; rifampicin-soaked 26 mm graft and subclavian reconstruction	Duration, arrest time and blood loss not reported. Required cardiopulmonary bypass, profound hypothermia, neurological monitoring and spinal-cord protection
Trimarchi et al.	Complete arch endograft explant through left thoracotomy using fourth- and seventh-intercostal access	Left carotid-subclavian bypass 2 days before explant; cardiopulmonary bypass; cooling to 20 °C; circulatory arrest; distal perfusion through the femoral artery; reconstruction with a self-made bovine xenopericardial tube; pedicled intercostal flap	Duration, arrest time and blood loss not reported. Required vascular and cardiothoracic surgical teams, intraoperative esophageal endoscopy and postoperative contrast testing
Hanif et al.	Complete explant using a staged extra-anatomical approach	Right axillobifemoral bypass first; explant 48 h later through left thoracoretroperitoneal access; cryopreserved human allograft; SMA and bilateral renal reconstruction; reinforced aortic stump; triple omental flaps and antibiotic beads	Duration and blood loss not reported. Temporary thoracoabdominal closure and additional debridement the following day
Panettella et al.	Complete explant through median laparotomy with supratruncal exposure and Mattox maneuver	Supratruncal clamping for 15 min, changed to transrenal clamping for 40 min; reconstruction using a hand-sewn bovine pericardial tube with biosynthetic iliac limbs; preattached saphenous-vein renal conduits were prepared but ultimately unnecessary; omentoplasty	8 h. Required massive transfusion: 11 red-cell and 1 platelet concentrates plus coagulation products. Temporary VAC closure and several second-look procedures
Bugna et al.	Complete explant through a staged extra-anatomical approach	Rifampicin-soaked axillobifemoral bypass first; second stage 24 h later through midline laparotomy; cryopreserved aortoiliac allograft; celiac, SMA, bilateral renal and left hypogastric reconstruction; reinforced aortic stump with fascia lata and omental coverage	Duration not reported. Blood loss: 1800 mL, of which 750 mL was recovered by cell salvage. Transfusion was not accepted because of the patient’s Jehovah’s Witness faith

## Data Availability

No new data were created or analyzed in this study. Data sharing is not applicable to this article.

## Supplementary Materials

The following supporting information can be downloaded at: https://www.mdpi.com/article/10.3390/medsci14050574/s1, Supplementary File S1: Complete data set; Figure S1: anatomical and topographical constraints during complex aortic endograft explanation; PRISMA 2020 Checklist.

## Abbreviations

The following abbreviations are used in this manuscript:

bEVAR	Branched Endovascular Aortic Repair
fEVAR	Fenestrated Endovascular Aortic Repair
chEVAR	Chimney Endovascular Aortic Repair
CTA	Computed Tomography Angiography
CT	Computed Tomography
LOS	Length of Stay
EVAR	Endovascular Aortic Repair
FDG-PET/CT	Fluorodeoxyglucose Positron-Emission Tomography/Computed Tomography
SMA	Superior Mesenteric Artery
JBI	Joanna Briggs Institute
CARE	CAse REport guideline
ESVS	European Society for Vascular Surgery
CRP	C-Reactive Protein
NLR	Neutrophil-to-Lymphocyte Ratio
SIRI	Systemic Inflammation Response Index
